# Audiovestibular Symptoms at the Intensive Care Unit: A Narrative Review

**DOI:** 10.7759/cureus.18421

**Published:** 2021-10-01

**Authors:** Luisa M Escobar, Melissa Castillo-Bustamante, Marco Gonzalez

**Affiliations:** 1 Critical Care Medicine, Medical School, Health Sciences School, Universidad Pontificia Bolivariana, Medellín, COL; 2 Otolaryngology, Medical School, Health Sciences School, Universidad Pontificia Bolivariana, Medellín, COL; 3 Otolaryngology - Head and Neck Surgery, Massachusetts Eye and Ear Infirmary, Boston, USA

**Keywords:** critical care, intensive care unit, hearing loss, tinnitus, vertigo diagnosis

## Abstract

Vertigo, tinnitus and hearing loss are the most common audiovestibular symptoms detected in the emergency departments and outpatients settings. However, little is known about these on patients at the intensive care unit. Although these symptoms may be common in this scenario, few studies have documented their onset, triggers and other factors associated to their presentation. The evaluation of these symptoms is a challenge for intensive care unit physicians, neurologists and otolaryngologists due to several factors as consciousness, systemic comorbidities, prolonged immobility and antibiotic therapy. The frequency of audiovestibular symptoms at the intensive care unit and the related events and factors associated to their presentation will be explored in this review.

## Introduction and background

Hearing loss, tinnitus and vertigo are the most common audiovestibular symptoms, which can affect quality of life, emotional, cognitive, and functional development [1,2]. These conditions have been widely treated on emergency and outpatients’ departments, where estimated prevalence found for hearing loss, vertigo and tinnitus are 6%, 1% and 11.9%, respectively [3-5]. Clinical evaluation of audiovestibular symptoms in these settings include physical examination, auditory and vestibular testing, as well as diagnostic and follow-up questionnaires, based on directed protocols and worldwide consensus [6-8]. However, the bedside and complimentary evaluation of these symptoms in some scenarios as the intensive care unit are limited and may represent a challenge for providers.

Individuals in the critical care unit are exposed to several environmental, biological and mechanical factors that may affect hearing and balance [9]. Some of these include noise, ototoxic medications, sepsis, electrolyte abnormalities, dehydration and malnutrition [10-15]. Others described include exposition to led monitors, ventilation equipment, changes on the ventilation and vital capacity, limited body movements, and medical complications associated to systemic patient’s disorders [16]. However, limited data and studies are focused on describing how these audiovestibular symptoms are found in patients at the intensive care unit.

Herein, we performed a narrative literature review to determine the frequency of audiovestibular symptoms at the intensive care unit and the related events and factors associated to their presentation. 

## Review

Methods

This narrative review was conducted between August and September 2021. We used the MeSH headings “vertigo”, “hearing loss”, “tinnitus”, “intensive care unit”, and “critical care”, to search PubMed, Embase, and Google Scholar databases for case reports, case series, retrospective chart reviews, prospective studies, cohort and case-control studies. This search aimed to (1) describe the frequency of audiovestibular symptoms at the intensive care unit and, (2) describe the audiometric and vestibular testing findings in patients who complained of audiovestibular symptoms at the intensive care unit.

The search was limited to articles between 2001 and the year 2021. We included complete manuscripts published in English which described audiovestibular findings such as vertigo, tinnitus, and hearing loss at the intensive care unit in populations over 18 years old; hospitalized patients at the intensive care unit who described the onset of audiovestibular symptoms at the intensive care unit, manuscripts which described the frequency of audiovestibular symptoms in populations during intensive care unit stay. Studies that included audiovestibular symptoms with SOFA (the Sequential Organ Failure Assessment) score assessment were also included (Score used for quantification of the number and severity of failed organs) [17].

Studies outside the field of Otolaryngology and Intensive Care Medicine were excluded. Articles related to COVID-19 studies and audiovestibular symptoms were also excluded, as they are not the goal of this review and the pathogenesis and follow-up of these symptoms are still being an understudy. Articles focused on pediatric populations and neonatal intensive care units were excluded. Results were cross-checked among the three authors. In total, 216 indexed papers were obtained in the initial search. From these, only seven indexed articles were selected because they reported patients with audiovestibular symptoms at the intensive care unit. The quality of evidence in the published articles was reviewed according to the 2009 Levels of Evidence of the Oxford Centre for Evidence-Based Medicine.

There were seven studies reviewed (two case reports, three prospective studies, and two retrospective chart reviews) including 571 patients with audiovestibular symptoms such as dizziness (D), vertigo (V), hearing loss (HL), and tinnitus (T), who were observed and followed-up at the critical care unit. (Table 1).

**Table 1:** Studies reporting audiovestibular symptoms at the intensive care unit.

Article	Type of study	Country	N	D	V	HL	T
Hamill-Ruth, 2003 [16]	Prospective	United States	442	-	-	+	-
Friedman, 1975 [18]	Case Report	United States	2	-	-	+	-
Hamill-Ruth,1998 [19]	Prospective	United States	17	-	-	+	-
Johansson,2012 [20]	Prospective	Sweden	13	-	-	+	+
Fujiwara, 2021 [21]	Retrospective	Japan	71	-	-	+	-
Dao-Ming, 2019 [22]	Retrospective	China	25	+	+	-	-
Burrows, 2018 [23]	Case Report	United Kingdom	1	-	+	-	-

Review

The age average of studies included was 60.8 ±8.1 with a range between 23-84.8 years. Males (52.2%%) presented more frequent admission to the intensive care unit than females. Audiovestibular symptoms were more often present in males (33.5%) on the case reports, retrospective and prospective studies included.

The median length of stay average at the intensive care unit was reported between 2 and 4 days with ranges between 1 and 16 days in three studies. Only one prospective study indicated an intensive care unit length of stay of 16.4 days with no estimated range. The median length of stay in the hospital was reported in two retrospective studies, indicated mean averages of 17 (range 4-71) and 10 (range 3-59) days. SOFA score found in two retrospective chart reviews was 2.70 ± 2.42 and 3.4 ± 1.8. Multiple trauma, sepsis, systemic organ failure, postoperative management, trauma, cardiovascular disease, and pulmonary diseases included the main etiologies for admission at the intensive care unit in most of all studies included.

Five studies (one case report, one retrospective study, and three prospective studies) revealed hearing loss as one of the most common audiovestibular symptoms in patients at the intensive care unit [16,18-21]. The age average of these studies was 50.5± 9.3 years. Men were mainly affected (60%). Primary causes of admission to critical care unit in patients with hearing loss included multiple trauma, postoperative monitoring, sepsis, organ transplant, single system failure, Guillain-Barré and, cardiovascular and pulmonary disorders. The median length of stay was reported between 2 and 16.4 days.

Hearing loss was associated with manual respirator use and increased positive pressure while ventilating in two cases, while in another case in one prospective study presented hearing loss without ventilator use (Table 2). In this case, thrombosis was reported in the chart review. Evidence of otoscopy was described in one prospective study, where cerumen impaction (30.9%) drainage (6%), trauma (7%), tympanic membrane perforation (0.4%), and external auditory canal stenosis (0.6%) were found in hospitalized patient at the intensive care unit with suspicion of hearing loss. Audiologic testing was performed in two prospective studies, describing higher presentations of curves type A and C in patients with shorter length of stay and curves type B in patients with long critical care unit stay. Distortion product otoacoustic emission (DPOAE) was reported in two prospective studies. Abnormal DPOAE results were obtained in 77.8% of patients with suspected hearing loss. The failure rate of DPOAE was highly seen in males than females (61.5% vs 48.6%).

The mean age of passing DPOAE was significantly lower than those failing (44.2 ± 16.4 vs 66 ±16.6). Higher failure rates (38-70%) of DPOAE were reported in patients with cardiopulmonary disorders such as aortic aneurysm, pulmonary embolism, pneumonia, and pulmonary and cardiac failure. No other audiologic testing was reported in the selected studies (Table 2).

**Table 2:** Hearing loss at the critical care unit.

Article	Type of study	N	Mean age	Mean length of stay (days)	Primary causes admission	Otoscopy finding	Auditory testing
Hamill-Ruth, 2003 [16]	Prospective	442	54	-	Multiple trauma Postoperative monitoring Sepsis Organ transplant Single system failure	Cerumen impaction Drainage Trauma Tympanic membrane perforation External auditory canal stenosis	Tympanograms: Type A (short-term admission) Type C (short-term admission) Type B (long-term admission) DPOAE: Mean Age Passing 44.2 ± 16.4 Mean Age Failing vs 66 ±16.6 Failure rate: Females 48.6% Males 61.5%
Friedman, 1975 [18]	Case Report	2	29	1	Case 1: Cor pulmonale Case 2: Cardiorespiratory arrest	Perilymph fistulae	Pure tone audiometry: Case 1: Sensorineural hearing loss 60 dB air-bone gap at all frequencies Word score: 90% Case 2: Right sided sensorineural hearing loss Word score: 6%
Hamill-Ruth,1998 [19]	Prospective	17	-	4	Not described	Not described	DPOAE: Initial Screening: 70.8% Abnormal After discharge from critical care unit: Abnormal 77.8% After discharge from the hospital: 44% Auditory brainstem responses: Thresholds >or=to 50dB: Initial screening 38.9% After discharge from critical care unit: 61.1% After discharge from the hospital: 50%
Johansson, 2012 [20]	Prospective	13	66.3	9.84	Pneumonia Sepsis Aortic aneurysm Guillain-Barré Whipples Surgery Pancreatitis Thrombosis One case with hearing loss associated to thrombosis. Length of stay for this patient 3 days	-	-
Fujiwara, 2021 [21]	Retrospective	71	72.5	2	Infectious disease Bleeding Metabolic disease Cardiovascular disease Postoperative management Pulmonary disease Trauma	-	Pure tone audiometry: Changes documented 4.14 ± 5.93 dB in women 4.03 ± 8.60 dB in men

Sensorineural hearing loss was only reported in one retrospective study [22]. Primary causes for intensive care unit admission included infectious disease, bleeding, metabolic disease, cardiovascular disease, postoperative management, pulmonary disease, and trauma. The median length of stay at the intensive care unit was 2 days (range 1-16). SOFA score reported was 2.70 ± 2.42. At least 64.8% of patients admitted to the intensive care unit had an emergency admission due to infectious and metabolic disorders. Pure tone audiometry (PTA) was performed before intensive care admission and after discharge. Changes in the PTA documented were 4.14 ± 5.93 dB in women and 4.03 ± 8.60 dB in men. 67.2% of patients admitted reported on the PTA an air-bone gap difference of 30 dB and above (Table 2).

Vertigo was reported in one case report and one retrospective study [23,24]. A male patient 61 years, with C3 complete spinal injury with benign paroxysmal positional vertigo, underwent repositioning maneuvers. No other associated factors for this vertigo such as vitamin D deficiency, hormonal dysfunction, head injury, or skull base fracture were documented. On the retrospective chart review, the age average was 60 years (range 23-81). Males were more affected than females (1.8:1). Primary etiologies associated with intensive care unit admission were focal intracerebral hemorrhage, septic shock, arteriosclerosis, and cardiogenic cerebral infarction. SOFA score average was 3.4 ± 1.8. The median stay at the critical care unit was 2 (1-16) days. Primary vestibular findings reported in this study were dizziness (56%) and vertigo (44%). Accompanying symptoms and physical exam findings were nausea and vomiting (84%), balance disorders (56%), and nystagmus (0.9%). Neither specific vestibular testing results nor HINTS (Head-Impulse, Nystagmus, Test-of-Skew) [25] examination protocol used for patients with acute vertigo reports were described (Table 3).

**Table 3:** Other audiovestibular symptoms at the intensive care unit.

Article	Type of study	N	Main Audiovestibular Symptom	Mean Age	Mean Length of stay (days)	Primary Causes Admission	SOFA Score (Mean)	Symptoms associated	Auditory testing
Johansson, 2012 [20]	Prospective	13	Tinnitus	66.3	1	Pneumonia Sepsis Aortic aneurysm Guillain-Barré Whipples Surgery Pancreatitis Thrombosis One case with tinnitus associated to ventilation use. Diagnosed with aortic aneurysm	-	-	.
Dao-Ming, 2019 [22]	Retrospective	25	Vertigo	60	2	Focal cerebral Spontaneous intracerebral hemorrhage Septic shock Arteriosclerosis Cardiogenic cerebral infarction	3.4	Debutant symptoms: Dizziness (56%) Vertigo (44%) Accompanying symptoms: Nausea and vomiting (84%) Balance disorders (56%) Nystagmus (0.9%)	-
Burrows, 2018 [23]	Case Report	1	Vertigo	61	-	C3 complete Spinal injury	-	None	-

Tinnitus was only reported in a single patient in one prospective study [21]. This patient-reported an intensive care unit stay of 3 days. An aortic aneurysm was detected during his stay. Prolonged ventilator use was reported. No other complimentary information was given for this case (Table 3).

Discussion

To our knowledge, this is one of the first narrative reviews about audiovestibular symptoms at the intensive care unit. Although case reports, retrospective, and prospective studies revealed few data concerning the clinical presentation, primary causes of admission and changes on the auditory testing, further extensive clinical and experimental studies should be done to characterize the onset and progression of symptoms, audiovestibular changes on testing during and after discharge from the intensive care units.

Regarding audiovestibular symptoms at the intensive care unit, clinical studies described the higher presentation of hearing loss in patients with cardiopulmonary disorders and sepsis. Conditions such as tympanic membrane perforations, cerumen, and effusions in the middle ear were found as contributors of hearing impairment, supported by auditory testing with non-altered tympanometries on short stays and pathologic tympanometric curves in long-term stays. DPOAEs and ABRs showed an altered auditory pathway, which was positively correlated to clinical findings.

Sensorineural hearing loss was associated with infectious and metabolic diseases with established changes on the pure tone audiometry above 30 dB. Vertigo and dizziness were reported in short intensive care unit stay associated to autonomic symptoms and mainly presented in patients with multisystemic failure and septic shock. Tinnitus was only reported in one case with thrombotic disorders.

Audiovestibular symptoms present a challenge for intensive care physicians, otolaryngologists, and healthcare allies. Common mechanisms associated with hearing loss in in-patient scenarios include microemboli, perfusion abnormalities, hypercoagulability, and ototoxic drugs [18,26]. In addition to these, on the peri-intensive care period, several health practices may be involved in the pathogenesis of audiovestibular symptoms [27]. The use of endotracheal, nasotracheal, and positive pressure face or nasal masks, would potentially compromise the function of eustachian tubes leading to ascending bacterial colonization, changes in the middle ear pressure, obstruction, and effusion in the middle ear, leading to conductive hearing loss [27,28]. Other healthcare practices may simulate conductive losses such as inadvertent earwax removal with drops, cleansing solutions, clotted blood, or foreign bodies [18]. Other conductive hearing losses documented at critical care units are tympanic membrane perforations and disruption of the ossicular chain associated with temporal bone fractures [27,28].

Sensorineural hearing loss in this narrative scope was mainly found in patients who presented infectious diseases, bleeding, and trauma. The use of ototoxic medications such as antibiotics, antifungals, and chemotherapeutics may be associated with the inner and outer hair cells during a prolonged stay at the intensive care unit due to infectious diseases [28]. Some other infectious disorders at intensive care units are meningitis, which can also lead to ossification of cochleas and hearing impairment [28]. Head trauma and brain injury are usually associated with potential injury to the inner ear, as well as closed or penetrating head trauma and hemorrhagic lesions in the auditory cortex.

In this scope review, vertigo was associated with expansive lesions and potentially life-threatening events such as spinal injury, tumors, and hemorrhages which can affect vestibule centers and pathways due to proximity, enlargement, and vascular involvement [23,29]. Even though these events were acute and associated with central nervous system injury, other contributors may play role in the pathogenesis of vertigo at the intensive care unit such as prolonged immobility, head injury, skull base fractures, and previous vitamin d deficiency [30]. Further studies are in need to correlate these events in patients at the intensive care unit.

Tinnitus was reported only in one case at the intensive care unit. A cardiovascular disorder and prolonged noise exposition were reported as possible contributors, however other environmental and patient factors at the intensive care unit may be involved in this pathogenesis, contribute to its onset including head and neck injuries, ototoxic drug use, and noise exposition [30]. Although tinnitus would be reported, objective auditory testing and questionnaires for tinnitus qualification would benefit the characterization of tinnitus at the intensive care unit.

The main limitations of this study are the small number of studies included the lack of information on audiometric and vestibular testing, limited data regarding the type of vertigo and tinnitus and, unregistered clinical and audiometric follow-up.

Further clinical studies including self-report of audiovestibular symptoms are needed to understand the clinical progression of these. Also, the temporal bone histopathologic analysis will be critical to understanding middle and inner ear changes of patients who underwent intensive care units.

## Conclusions

Hearing loss, tinnitus, and vertigo are some of the audiovestibular symptoms reported on patients at the intensive care units. Altered auditory testing on acoustic immittance, ABR, and DPOAES were found in patients treated at the intensive care unit. Vascular, infectious, and systemic changes, as well as external factors proper of this clinical scenario, may contribute to the onset of these symptoms. Further studies are needed to understand the clinical progression of these symptoms and the clinical characteristics of each.
